# The Principles and Practice of Surgery

**Published:** 1890-07

**Authors:** 


**The Principles and Practice of Surgery**, being a Treatise on Surgical Diseases and Injuries. By D. HAYES AGNEW, M. D., LL. D., Professor of Surgery in the Medical Department, University of Pennsylvania. Profusely illustrated. Second edition — thoroughly revised — with additions. In three volumes. Imperial octavo. Vol. I., pp. 1114; Vol. II., pp. 1066; Vol. III. pp. 785. Philadelphia: J. B. Lippincott Company. London: 10 Henrietta street, Covent Garden. 1889. Price, $7.50 per volume.

The wonderful strides of surgery in the past ten years has rendered much of the literature of 1880 of little value, except for reference; or it has, at least, been necessary to rewrite much of it, and recast the lines of other much, until its complexion has been so changed as to make it difficult of recognition.

Agnew's first edition appeared in 1881, and the present one illustrates by comparison with it just the idea we would wish to convey in the foregoing paragraph; it is especially true as regards surgical treatment, and is partially true as pertains to the general scope and plan of all surgical treatises. The author of this one has been so well known to the professional world as a teacher and writer for thirty years or more that very little need, or indeed can, be said to add or detract from his work as recorded in these volumes. They speak for themselves in no uncertain way; they are the result of practical observations by a practical observer, and must forever stand as a classic in the literature of American surgery. When a man tells what he knows and describes what he has seen in simple language, it has a weight that no mere theorist can attain with ornate speech and flowery utterance. This is a period in medicine when the outcry is for facts, and whoever establishes a fact in the study of disease has done humanity a lasting service. It has fallen to the lot of Dr. Agnew to serve his fellow-men in the direction named in a manner such as but few can boast. He attained the ripest years of mature judgment before he ventured to speak to his professional brethren from the chair or in the attitude of an author, and he has waited until a fitting time to put forth a second edition of his first great work. It was wise to wait long ; it were unwisdom to wait longer. To enjoy the fruits of his active labor, and live in the midst of its fullest appreciation, ought to be an ample reward to any man. These are the crowning glories of Dr. Agnew, and we hope he may yet live long to enjoy them.

The illustrations in this work are particularly good, and amplify the text in a useful manner. The paper and press-work are such as might be expected to accompany such a treatise. It is a work that must necessarily find a place in the library of every surgeon, and we should consider our own sadly deficient without it.

